# The Autism Family Experience Questionnaire (AFEQ): An Ecologically-Valid, Parent-Nominated Measure of Family Experience, Quality of Life and Prioritised Outcomes for Early Intervention

**DOI:** 10.1007/s10803-017-3350-7

**Published:** 2017-11-18

**Authors:** Kathy Leadbitter, Catherine Aldred, Helen McConachie, Ann Le Couteur, Dharmi Kapadia, Tony Charman, Wendy Macdonald, Erica Salomone, Richard Emsley, Jonathan Green, The PACT Consortium, Barbara Barrett, Sam Barron, Karen Beggs, Laura Blazey, Katy Bourne, Sarah Byford, Rachel Cole-Fletcher, Julia Collino, Ruth Colmer, Anna Cutress, Isobel Gammer, Clare Harrop, Tori Houghton, Pat Howlin, Kristelle Hudry, Sue Leach, Jessica Maxwell, Jeremy Parr, Andrew Pickles, Sarah Randles, Vicky Slonims, Carol Taylor, Kathryn Temple, Hannah Tobin, George Vamvakas, Lydia White

**Affiliations:** 1 University of Manchester, Manchester, UK; 2 Newcastle University, Newcastle upon Tyne, UK; 3 Northumberland, Tyne & Wear NHS Foundation Trust, Newcastle upon Tyne, UK; 4 Institute of Psychiatry, Psychology & Neuroscience, King’s College London, London, UK; 5 Greater Manchester Mental Health NHS Foundation Trust, Manchester, UK; 6 University of Turin, Turin, Italy; 7 Manchester Academic Health Sciences Centre, Royal Manchester Children’s Hospital, Manchester, UK; 8 Social Development Research Group, University of Manchester, Room 3.312, Jean McFarlane Building, Oxford Rd., Manchester, M13 9PL UK

**Keywords:** Autism Spectrum Disorder, Family experience, Parent, Intervention, Quality of Life, Wellbeing

## Abstract

There is a lack of measures that reflect the intervention priorities of parents of children with autism spectrum disorder (ASD) and that assess the impact of interventions on family experience and quality of life. The Autism Family Experience Questionnaire (AFEQ) was developed through focus groups and online consultation with parents, and reflected parental priorities. It was then administered to the parents of children enrolled in the Pre-school Autism Communication Trial and its 6-year follow-up study. The AFEQ showed good convergent validity with well-established measures of child adaptive functioning, parental mental health and parental wellbeing. It was sensitive to change in response to a parent-mediated intervention for young children with autism, showing treatment effect at treatment endpoint which increased at six-year follow-up.

**Electronic supplementary material:**

The online version of this article 10.1007/s10803-017-3350-7) contains supplementary material, which is available to authorized users.

## Introduction

There is a notable paucity of parent-nominated measures designed to assess intervention outcomes for children with neuro-developmental disabilities, such as autism spectrum disorder (ASD), and their families (McConachie et al. 2015; Morris et al. 2014, 2015). Parents of children with ASD are often best placed to advocate for the interests and opinions of their children and to observe and report on their child’s progress, particularly when those children have significant intellectual and communicative disabilities and are unable to self-report (Morris et al. 2014). Many autism interventions, particularly in the pre-school years, are parent-mediated (e.g., Kasari et al. 2010; Oono et al. 2013; Pickles et al. 2016; Rahman et al. 2016) and effective services for the child with ASD can have “spillover effects” for the rest of the family (Payakachat et al. 2012). Parents therefore have a vital contribution to make on two levels: (1) in specifying which intervention outcomes are important for their child, for themselves and for their family and thereby setting the success criteria for interventions, and (2) in reporting on the measures of those outcomes for their individual children.

The measurement of patient- and parent/carer-centred outcomes has become an integral part of the evaluation of treatment effectiveness and cost-effectiveness in the UK and internationally (see, for example, the Promis System of the US National Institutes of Health 2017, and the NHS Outcomes Framework of the UK Department of Health 2017). This gives the clear mandate that patients and their families/carers should be central to the process of prioritising which outcomes matter most within any particular health domain and therefore determining the criteria by which interventions are judged (Marshall et al. 2005; Morris et al. 2015; National Institute for Health and Care Excellence 2013; Tait and Lester 2005). This mandate extends to paediatric research, with an emphasis on the crucial role of parents and carers in nominating and reporting when their children are not developmentally able to do so themselves (Morris et al. 2009).

Alongside the policy drive for patient- and parent/carer- nominated outcomes, there has recently been increased attention more generally to the research priorities of individuals with ASD themselves and their parents and carers (Iemmi et al. 2017; McConachie et al. 2015; Pellicano et al. 2014). Interventions to improve individual and family wellbeing and quality of life are frequently cited as a priority by people with ASD and their parents and carers (McConachie et al. 2015; Pellicano et al. 2014). Despite this, the inclusion of patient- or parent-nominated measures or, indeed, any outcome measure of quality of life in autism intervention research, and particularly within randomised controlled trials, is still uncommon (Burgess and Gutstein 2007; McConachie et al. 2015; Pellicano et al. 2014), with the emphasis firmly on child developmental outcomes such as intellectual ability, communication and language skills, play or autism symptomatology (e.g., Estes et al. 2015; Kasari et al. 2012; Kasari 2015; Pickles et al. 2016).

The measurement of quality of life in preschool children is complex (Eiser and Morse 2001; Grange et al. 2007). Standardised health-related quality of life indicators have been developed for childhood chronic conditions (Eiser and Morse 2001; Solans et al. 2008), such as the Pediatric Quality of Life Inventory (Varni et al. 2001), the EuroQol—5 Dimensional Questionnaire—Youth (Wille et al. 2010) and the Child Health Utility—9 Dimensions (Stevens 2012). Such measures are psychometrically strong and effective in providing a metric of general wellbeing in children with a range of conditions, particularly physical illness. They have less utility for measuring condition-specific dimensions. They do not, for example, capture the unique and complex practical, social and emotional intricacies of living with ASD (Eapen et al. 2014). The World Health Organisation has identified this gap and is currently developing a comprehensive Core Set for Autism Spectrum Disorder (ASD) within its International Classification of Functioning, Disability and Health (ICF), which aims to describe the lived experience of individuals with ASD across various health, wellbeing and developmental domains (Bölte et al. 2014; de Schipper et al. 2016).

Whilst child-specific quality of life is an important outcome within autism intervention work, a wider focus that encompasses the wellbeing of parents and the whole family system has been recommended, particularly within intervention aimed at young children (Hastings et al. 2014; Payakachat et al. 2012; Tint and Weiss 2016). The raised levels of fatigue, stress, anxiety and depression in parents of children with ASD are well documented (e.g., Dykens et al. 2014; Giallo et al. 2011; Quintero and McIntyre 2010). Siblings have also been shown to be at risk of higher levels of emotional and behavioural difficulties (e.g., Rodrigue et al. 1993; Ross and Cuskelly 2006). These effects are, of course, often multi-directional: sibling wellbeing can be related to parental coping and stress (Tsai et al. 2016) and parental stress and distress can affect child adjustment and behaviour, as well as vice versa (Hastings et al. 2014; Totsika et al. 2013; Zaidman-Zait et al. 2014). However, importantly, parents of children with ASD emphasise that there are many positive aspects to their family life (McConachie et al. 2015) and several beneficial effects of having a sibling with ASD have been reported empirically, such as reduced conflict in the sibling relationship (Kaminsky and Dewey 2002) and increased general resilience (Bayat 2007). A strengths-based approach to ASD has also been called for by researchers (Burnham Riosa et al. 2017; McCrimmon and Montgomery 2014). It would therefore be valuable to document family strengths, as well as difficulties.

Mental health constructs like parenting stress have been commonly utilised to tap parental wellbeing (e.g., Hayes and Watson 2013; Zaidman-Zait et al. 2014) and, more recently, quality of life indicators specific to parents of children with ASD have been developed, such as the Quality of Life in Autism Questionnaire (Eapen et al. 2014). However there is, to our knowledge, no parent-generated measure of family experience in the autism literature that can be used to assess the impact of an intervention. In this paper we describe the development of a parent-nominated measure of family experience, quality of life and prioritised outcomes for early intervention designed to address this measurement gap. This measure was developed and tested within the context of a large randomised trial of a pre-school parent-mediated intervention.

### Background: The PACT Intervention, Trial and Follow-Up

Pre-school Autism Communication Therapy (PACT Therapy) is a parent-mediated video-aided communication-focussed intervention for pre-school children with autism and their parents. The UK Medical Research Council Pre-school Autism Communication Trial (PACT Trial) was a two arm parallel group randomised controlled trial (RCT) of 152 pre-school children with core autism. It ran between 2006 and 2009 across three UK centres (Manchester, London and Newcastle) and evaluated the effectiveness of PACT therapy plus treatment-as-usual (TAU) versus TAU alone (Green et al. 2010; Pickles et al. 2015). A subsequent follow-up phase assessed outcomes at 6 years after the end of the treatment phase (Pickles et al. 2016). The trial was registered on International Standard Randomised Controlled Trial, number ISRCTN58133827, and the protocol is available at http://research.bmh.manchester.ac.uk/pact.

At trial entry, all children had a clinical diagnosis of autism and met criteria for core autism on the researcher-administered Autism Diagnostic Observation Schedule - Generic (Lord et al. 2000) and two of the three domains of the Autism Diagnostic Interview—Revised (Lord et al. 1994). 77% of the sample was not yet using regular, spontaneous phrase speech. The sample was ethnically and socio-economically diverse (57% white; 23% single parents; 74% with at least one parent with post-16 qualifications). The PACT treatment group consisted of 77 children (6 girls) aged 26–60 months (mean age = 45 months) with a mean non-verbal IQ age equivalence of 27 months. The TAU consisted of 75 children (8 girls) aged 24–60 months (mean age = 45 months) with a mean non-verbal IQ age equivalence of 25 months. Other demographic details of families were balanced across the arms at baseline. The trial showed a strong treatment effect in favour of the PACT intervention in observed parental synchronous responsive behaviours and child communicative behaviours in dyadic communication, and in parent-reported outcome ratings on child language and social communication. However, there were more modest effects on child social affective autism symptoms and on standardised objective tests of child language (Green et al. 2010). At follow up 80% of the sample was assessed (mean age 10.5 years; *SD* = 0.8; *n* = 121). Intention-to-treat analysis based on initial randomisation showed a reduction of autism symptom severity at treatment endpoint which was sustained to 6-year follow-up and represented a 17% relative reduction in symptom severity in the treatment arm (Pickles et al. 2016). The improvement in child dyadic communicative behaviours seen in the intervention group at trial endpoint was sustained into follow-up, but standardised measures of language development continued not to show group differences.

The commissioning and protocol of the PACT trial included a strategy to promote involvement of service users in pre-trial research design, and to address the measurement gap in relation to family experience outcome measures through the development of a parent-generated measure of child and family wellbeing for use in the trial. This new measure aimed to reflect both parental report of their personal and family experience, and their autistic child’s development and adjustment. The specific focus was on whether the parental priorities for change were achieved through the pre-school intervention. It was hypothesised that, as the PACT intervention works partly through changing the quality of parental responsiveness to child communication, it could result in more general positive changes in family life, and improved parental confidence and morale. Since the assessment had not been developed prior to the trial, data from the resulting instrument would not be used in the formal outcome analysis, but secondary post hoc analyses could contribute to the evaluation of this prototype of an autism specific, parent-nominated change measure that could be suitable for future intervention and other research.

## Methods

### Developing the Measure

A two-phase process was used. Firstly, a series of focus groups was held with parents of children with autism to generate a core set of outcome parameters. Secondly, we implemented a larger web-based consultation using the resources of the UK National Autistic Society (www.autism.org.uk) in order to subject the initial parameters to wider review, comment and refinement. Such a method is an extension of recommended methods for developing measures of this kind (e.g., Eiser and Morse 2001, p. 90).

#### Focus Group Phase

Thirty-one parents of pre-school or school-aged children with autism were recruited from local clinical services and parent-support groups and attended one of five focus groups (four held in Manchester UK and one in Newcastle UK; 4–11 participants in each group). The groups were convened and led by members of the PACT Principal Investigator team and an independent qualitative researcher (WM). The aims of the focus groups were to explore the specific parameters that parents identified as the most important outcomes from a pre-school communication intervention for autism. Following consultation with parents and stakeholders at the National Autistic Society, four key topics of enquiry were pre-specified: (a) parental personal life and relationships; (b) general family functioning (including sibling needs); (c) specific outcomes for the child with autism related to general development, communication and learning; (d) the child’s symptoms in terms of emotional wellbeing and behaviour. The initial orientating question for the groups was: ‘Given a communication-focused intervention for a child with autism in the preschool years, what would be the key aspects of family life, child behavior or development that—if they changed—would make you feel the intervention had been a success?’ Focus group leaders structured the groups to cover the pre-specified domains without directing the content.

#### Qualitative Analysis

Audio-tapes of each focus group meeting were transcribed for analysis using NUDIST software in a constant comparative method (Glaser and Strauss 1967). An initial thematic coding framework was devised and the transcripts analysed line-by-line and coded against the framework. Themes obtained were then discussed with the wider team and constant comparison was used to refine a final set of themes. Individual statements were then abstracted from these themes to serve as response items within the questionnaire. This process resulted in 78 questionnaire items. Examples of the themes and the abstracted questionnaire items are shown in Table 1.

**Table 1:** Examples of focus group themes and resulting questionnaire items

Domain	Theme	Questionnaire item
Parent	*Parental battle*: High levels of frustration from having to constantly ‘fight’ for support for their child	“It’s a continual battle to get the right help for my child”
	*Realistic expectations*: For many parents having realistic expectations about the rate of improvement they could expect from an intervention was important. There was general understanding that they needed to adjust to a new set of milestones for their child with autism. For some parents the slow rate of change was very difficult to come to terms with, whilst for other parents (particularly of older children) there was an appreciation of the slower rate of change and improvement they had observed in their child, often over years	“I have realistic milestones for my child’s development”
Family	*Wider family attitudes*: Some parents talked about the lack of acceptance and understanding of their child in the wider family and this lack of understanding making it difficult for them to attend extended family events and occasions	“I feel confident to go out to family events with my child”
	*Routines and structure at home*: There was discussion around the problems around transition between school and home and the problems because of a perceived lack of structure and ‘routine’ in the home environment	“I feel confident in making routines at home more manageable for my child”
Child development	*Changes in child’s social experience*: All parents wanted their child to be able to play with other children, to develop and keep friendships, and to be socially accepted and included. One key consensus marker of improved social experiences and relationships mentioned by several parents was if their child was invited to birthday parties	“My child gets invited to birthday parties”
	*Ability to communicate illness* A number of parents described the difficulty associated with their child’s inability to understand physical symptoms, what is going on in their body and to communicate physical symptoms to others	“I know when my child feels poorly”
Child symptoms	*Emotional development*: Parents were looking for strategies that could help their child to understand and express their feelings and as a parent to understand their child’s feelings in order to help them to be happier and more relaxed	“My child is happy”
	*Family outings*: A recurring theme was the desire to be able to go out as a family without being overwhelmed by feelings of embarrassment	“My child is embarrassing when going out”

#### Website Consultation

The 78 items derived from these focus group themes were uploaded in the form of a draft questionnaire onto an online survey facility within the website of the UK National Autistic Society. This website facility allowed the presentation of the items of the draft questionnaire, contextualised with an introduction about its aims. Respondents rated each question against two criteria: (1) *Clarity*: comprehensibility and lack of ambiguity; (2) *Usefulness* of the question for evaluating the effectiveness of a communication treatment for autism. The website consultation was live over a 10-day period in February/March 2006. Over this period, 35 completed questionnaire responses by parents of children with autism were received and collated by staff at the National Autistic Society.

#### Analysis of Ratings

The web consultation resulted in weighted ranks against both criteria (i.e., clarity and usefulness) for each of the items presented. Items rated in the bottom quintile for relevance were discarded. Items weighted highly for relevance but low for clarity were retained but re-worded, or discarded if it was difficult to rephrase the item into a short and simple statement that was clearer.

### Data Preparation Within the PACT Trial and Follow-up

The resulting questionnaire had 56 items. Items included both positively and negatively worded statements and were scored on an order scale: 1 = always to 5 = never, with an option for “Not Applicable”. Items were organised into domains which matched onto the pre-specified focus-group topics: (1) experience of being a parent of a child with autism; (2) family life, (3) child development (development, understanding and social relationships); and (4) child symptoms (feelings and behaviour). This questionnaire was then rated by parents, with researcher support if requested, as part of the baseline and endpoint assessments of the PACT trial. Prior to group unblinding and analysis, this data was then subject to initial data cleaning. Eight items that were rarely endorsed were excluded, including items on language (when many children were non-verbal), and siblings (when there often were none). The resulting 48 item questionnaire was named the Autism Family Experience Questionnaire (AFEQ; Online Appendix I). Items were organised into the four pre-specified domains; Table 2 shows how items map onto these domains. The AFEQ included both positively and negatively worded statements and was scored on an order scale: 1 = always to 5 = never, with an option for “Not Applicable”. The 48-item AFEQ was then used in the PACT 6-year follow-up study.

**Table 2:** Autism family experience questionnaire domains and items

Domain	Items
Experience of being a parent	1–13
Family life	14–22
Child development, understanding and social relationships	23–36
Child symptoms (Feelings and behaviour)	37–48
Total AFEQ Score	1–48

### Data Analysis

We planned an initial descriptive analysis of the pattern of AFEQ scores across the three time-points and an examination of the internal consistency of the domain scores and total score. *A pre-specified analysis plan* of the AFEQ data (included in the original trial proposal and protocol) included a study against an external criterion referent for the child development domain of the AFEQ, using the parental Vineland Adaptive Behavior Scales, Second Edition (VABS; Sparrow et al. 2006), a well-validated parent-rated scale of child adaptive functioning. An additional analysis was planned within the follow-up phase of the trial to assess the convergent validity of the parent domain of the AFEQ with a study of the association of the parent domain with a well-established measures of adult mental health—the General Health Questionnaire-12 Items (GHQ-12; Goldberg 1992)—and of adult wellbeing—the Warwick-Edinburgh Mental Wellbeing Scale (WEMWBS; Tennant et al. 2007).

*The initial pre-specified analysis plan* included the examination of the change in AFEQ over time between treatment and TAU groups. For this treatment effect estimate we postulated a priori hypotheses based on the nature of the intervention and potential effects on aspects of family experience contained within the AFEQ. Thus we predicted that, compared to TAU, the PACT intervention would result in relative improvements at trial endpoint and at 6-year follow-up in: (1) the total AFEQ score; (2) each of the four domain scores (parent, family life, child development and child symptoms).

We compared baseline, endpoint and follow-up AFEQ total and domain scores between groups using an intention-to-treat approach, using the same analytic model as in the main analysis (Green et al. 2010; Pickles et al. 2016): each outcome was analysed separately using linear regression (analysis of covariance) including baseline measures of the outcome as covariate. In line with the main analyses we adjusted for the same set of baseline covariates as fixed effects, namely: age group (≤ 42 months, > 42 months), sex, centre (Manchester, London, Newcastle), non-verbal ability (mean non-verbal age-equivalent on the Mullen Scales of Early Learning; Mullen 1995), socioeconomic status (dichotomised as at least one parent with post-16 qualifications versus all others) and parental education (dichotomised as at least one parent in professional or administrative occupation versus all others). These were variables pre-specified as potentially having an effect on outcome scores independent of treatment.

## Results

### Descriptive Analysis of AFEQ and Internal Consistency

Of the total sample of 152 participants, AFEQ data were obtained from 145 parents at baseline assessment (7 participants had completely missing data), from 140/145 (97%) parents at trial endpoint (13 months later; 12 completely missing data), and from 105/145 (72%) parents at follow-up, 6 years from end of treatment. There were no significant demographic differences between the baseline sample and those for whom we had follow-up data. Total AFEQ scores were derived from these data. Individual items that were missing or entered as NA were pro-rated with mean scores of all items. Items that were negatively worded were reverse scored, so that throughout the data a lower score indicates a positive outcome, and a higher score is a poor outcome (minimum possible score is 48, maximum possible is 240).

At baseline, the total score for the sample as a whole had mean 141.0 (*SD* = 21.3), median 141.0 (range 81.8–188.0). At endpoint, the whole sample total score had mean 133.0 (*SD* = 22.8), median 134.4 (range 64.0–180.0). At follow-up, the total score had a mean 132.5 (*SD* = 24.6), median 133 (range 78.1–196.2). Therefore there was a slight reduction in scores between baseline and endpoint and stability on scores between endpoint and follow-up.

In order to assess the internal consistency of the 48 item questionnaire, we examined the scale reliability based on Cronbach’s alpha for the domain scores and the AFEQ total score, calculated from baseline data. All domains and the total score demonstrated excellent reliability at baseline: parent (alpha = 0.85), family (0.83), child development (0.81), child symptoms (0.79), AFEQ total (0.92).

### Comparative Analysis Against Parental VABS

For comparison and to assess the external criterion validity of the AFEQ, we correlated the scores on the AFEQ child development domain (items 23–36; 14 items) with the VABS total score at baseline, endpoint and follow-up. Both AFEQ and VABS were completed with 143 families at baseline (2 missing VABS in addition to the 7 missing AFEQs); 134 at endpoint (6 missing VABS in addition to the 12 missing AFEQs); and 102 at follow-up (3 missing VABS in addition to the 47 missing AFEQs). Recalling that a positive outcome is indicated by a low score on the AFEQ and a high score on the VABS, we would anticipate a significant negative correlation to indicate that the scales were producing similar results. The correlation between the VABS and child developmental AFEQ subtotal at baseline was *r* = − 0.478 (*p* < .001, *n* = 143), at endpoint was *r* = − 0.575 (*p* < .001, *n* = 134), and at follow-up was *r* = − 0.710 (*p* < .001, *n* = 102), indicating a moderate to strong association between the two measures at each of the three time-points.

### Comparative Analysis Against Parental Mental Health and Wellbeing Measures

To assess the external criterion validity of the parent domain of the AFEQ, we tested for an association between the parent domain score (items 1–13) and the GHQ-12 and WEMWBS total scores at trial follow-up. These adult mental health and wellbeing scales were not used as part of the trial baseline or endpoint assessment battery, so these analyses could not be conducted. The GHQ-12 total score was computed using the ‘GHQ scoring method’ (items scored 0-0-1-1; Goldberg 1992), with missing items recoded as low scores, resulting in a total score between 0 and 12, where a higher score indicates poorer mental health. Both the AFEQ and the GHQ-12 were completed by 101 parents (4 missing GHQ-12 questionnaires in addition to the 47 missing AFEQs). The correlation between the parent domain score and GHQ-12 was Spearman’s *Rho* = 0.408 (*p* < .001, *n* = 101; a Spearman’s rank correlation was conducted as the GHQ-12 distribution was highly positively skewed). The WEMWBS total score was the sum of scores on all 14 questionnaire items, with missing data on individual items pro-rated, resulting in a total score ranging from 14 to 70 where a higher score indicates increased wellbeing (Tennant et al. 2007). Both the WEMWBS and the AFEQ were completed by 103 parents (2 missing WEMWBS questionnaires on top of the 47 missing AFEQs). The correlation between the AFEQ parent domain score and the WEMWBS total score was *r* = − 0.528 (*p* < .001, *n* = 103).

### Treatment Effect Estimation

The results of the treatment effect estimation analysis (mean differences and effect sizes [Cohen’s *d*]), along with summary statistics of the total and domain scores, are shown in Tables 3 and 4.

**Table 3:** Summary statistics on AFEQ total and subscale scores for the PACT treatment and treatment-as-usual groups at baseline, endpoint and 6-year follow-up

AFEQ Domain Mean (SD)	PACT intervention group			Treatment-as-usual group		
	Baseline n = 76	Endpoint n = 72	Follow-up n = 51	Baseline n = 69	Endpoint n = 68	Follow-up n = 54
Total score	140.92 (21.78)	130.36 (23.59)	128.80 (28.11)	141.04 (21.00)	135.76 (21.66)	136.00 (20.52)
Parent	35.58 (7.62)	30.81 (7.28)	31.81 (7.72)	33.77 (6.76)	30.96 (7.08)	33.31 (7.91)
Family	26.07 (6.08)	25.18 (6.57)	23.37 (7.09)	25.65 (6.12)	25.54 (6.53)	25.02 (6.00)
Child development	44.68 (8.15)	41.27 (7.75)	39.72 (10.79)	46.43 (7.58)	44.44 (8.53)	42.37 (8.01)
Child symptoms	34.59 (5.82)	33.10 (6.29)	33.89 (5.49)	35.19 (5.71)	34.83 (5.84)	35.30 (4.93)

AFEQ low score = more positive outcome

**Table 4:** Treatment effect estimates

AFEQ domain	Endpoint			Follow-up		
	Mean difference (SE), p value	95% CI	Effect size 95% CI	Mean difference (SE), p value	95% CI	Effect size 95% CI
Total score	− 6.22 (3.12), 0.048	− 12.39, − 0.06	− 0.29 − 0.58, − 0.00	− 10.44 (4.90), 0.036	− 20.18, − 0.71	− 0.49 − 0.95, − 0.03
Parent	− 1.44 (1.08), 0.185	− 3.57, 0.07	− 0.20 − 0.49, 0.01	− 3.87 (1.64), 0.021	− 7.14, − 0.60	− 0.53 − 0.98, − 0.08
Family	− 0.88 (0.92), 0.342	− 2.69, 0.94	− 0.14 − 0.43, 0.15	− 2.42 (1.34), 0.075	− 5.09, 0.25	− 0.38 − 0.81, 0.04
Child development	− 2.20 (1.10), 0.048	− 4.38, − 0.02	− 0.28 − 0.55, − 0.00	− 3.02 (1.78), 0.094	− 6.56, 0.52	− 0.38 − 0.83, 0.07
Child symptoms	− 1.80 (0.91), 0.050	− 3.60, 0.00	− 0.31 − 0.63, 0.00	− 1.36 (1.07), 0.207	− 3.48, 0.76	− 0.24 − 0.60, 0.13

Negative coefficient = more beneficial effect for PACT compared to TAU

On the 48 item AFEQ total score, there was a statistically significant improvement of PACT over TAU at both trial endpoint and at 6-year follow-up, with a stronger effect at follow-up than at endpoint (Cohen’s *d* = − 0.49 and − 0.29, respectively). The treatment effects on the family domain score were non-significant at both endpoint and follow-up. On the parent domain there was a significant and moderately strong treatment effect at follow-up (*d* = − 0.53), which was not seen at trial endpoint. On the child development domain we found a significant effect of the treatment at endpoint (*d* = − 0.28), which was not sustained six years later and on the child symptoms domain we found a marginal effect at endpoint (*d* = − 0.31), also not sustained at follow-up.

## Discussion

The aim of the study was to develop a parent-generated measure of family outcomes in the context of a RCT of a parent-mediated video-aided pre-school communication-focused intervention for young children with autism (the PACT trial). In keeping with the patient- and parent/carer-centred outcome agenda and the research priorities of people with ASD and their families (Iemmi et al. 2017; McConachie et al. 2015; Pellicano et al. 2014), the AFEQ is truly user-generated. Our procedure started prior to the trial itself with focus groups and an online consultation with parents of children with ASD to generate parents’ own formulations of the priorities for early intervention outcomes. Participating parents were asked to consider the key outcomes under four domains: for themselves as parents, for their family as a whole, for their child’s development, and for their child feelings and behaviour (child symptoms). Thematic analysis generated themes which were formed into statements to serve as questionnaire items. The final selection of items was made using parental ratings of clarity and usefulness collected in the online consultation.

We conclude that such a process is feasible and productive and has culminated in a viable, ecologically-valid instrument with good internal consistency. Parents within the trial reported that they found the measure easy to complete and that they valued the opportunity to report real-life experiences for their children and other family members on metrics nominated by other parents of a child with ASD. These anecdotal reports are consistent with the findings that individuals with ASD and their parents and carers value wellbeing and quality of life as outcomes measures within intervention research (McConachie et al. 2015; Pellicano et al. 2014).

The importance of considering external validity in trials has been highlighted in the field of autism (Jonsson et al. 2016) and includes the extent to which new skills carry over into everyday life and valued outcomes. As an initial test of external validity we undertook a planned a priori analysis of the relation to the parent rated Vineland Adaptive Behavior Scales (VABS; Sparrow et al. 2006). This widely-used measure relates to child adaptive behaviour in a number of domains and thus was an obvious comparator for the AFEQ child development domain. There was a high correlation between the AFEQ and the VABS at each of the three timepoints of the PACT trial, in keeping with our hypothesis in the trial protocol that the parental VABS would act as an initial external validation of the child development domain of the newly developed instrument. A further test of the convergent validity of the AFEQ was the analysis of the association of the AFEQ parent domain and well-established measures of adult mental health - the General Health Questionnaire (GHQ-12; Goldberg 1992)—and wellbeing—the Warwick-Edinburgh Mental Wellbeing Scale (WEMWBS; Tennant et al. 2007), collected during the follow-up phase. A significant correlation with these measures suggested that the newly developed domain was tapping a construct that is linked to parental mental health and wellbeing.

An interesting point of note from the descriptive analysis was that the AFEQ total score taken across the two groups showed a slight reduction (improvement) between baseline and endpoint and then remained fairly stable to follow-up. The pattern of domain scores across timepoints shown in Table 3 suggests that this pattern is driven mainly by: (1) reductions in the parent and family domain scores in the PACT group only, and (2) decreases in scores in the child development domain across both groups, the latter perhaps reflecting developmental change across childhood.

A planned analysis within the trial protocol was to test the estimation of treatment effect based on the AFEQ. We found that the AFEQ total score showed a significant treatment effect at the 13-month trial endpoint and at 6-year follow-up, with a larger effect size at follow-up than at endpoint. These findings provide evidence that the AFEQ total score is sensitive to change in the expected direction in response to a parent-mediated intervention for young children with autism. They suggest that the AFEQ is tapping short and longer term benefits from the PACT intervention that extend beyond, and complement, the standardised observational and parent-report measures of child development and functioning reported in our main papers (Green et al. 2010; Pickles et al. 2016). A treatment effect was also seen on the child development domain at endpoint, consistent with the other positive parental report of outcomes on child social communication and language development at endpoint reported in our main paper (Green et al. 2010).

At follow-up, the parent domain showed a significant and moderately strong treatment effect; the family, child development and child symptoms domains showed non-significant changes over this time period. The six-year improvement in the parent domain seen in the treatment group is particularly illuminating, as this domain does not directly address aspects of the child’s functioning and wellbeing, but instead aspects of the parent’s coping, confidence and self-efficacy. We interpret this as showing that early parent-mediated intervention of this kind with relatively low intensity can bring about such sustained reported benefits on parental experience and morale years later. This adds weight to the notion that effective early intervention may lead to spillover effects for the wider family (Payakachat et al. 2012). It is also intriguing that the AFEQ parent domain showed a treatment effect given that the PACT therapy did not have a protective effect on parental mental health and wellbeing, over and above other child and family risk factors, when measured with established instruments (GHQ-12 and WEMWBS) and given that the AFEQ is well-correlated with these measures. As intended by its method of development, it may well be that the AFEQ items taps constructs that are more directly and specifically influenced by a parent-mediated intervention (e.g., items such as “I feel listened to by professionals” and “I feel I know how to help my child progress”), compared to the more general mental health and wellbeing constructs assessed by the GHQ-12 and the WEMWBS. The AFEQ may therefore be more sensitive to change resulting from an autism intervention, compared to more general questionnaires.

To balance its ecological validity as a parent-nominated measure, the AFEQ is completed unblind to treatment allocation (as is inevitably the case in psychosocial trials of this kind) and thus is subject to expectation effects that can substantially inflate estimates of treatment effect, compared to trials which use blind-rated outcomes (Sonuga Barke et al. 2013). The strength of the correlations between AFEQ and other questionnaire measures (GHQ-12 and WEMWBS) may be subject to common rater biasing, as they were often completed at the same session; the VABS, on the other hand, was completed as an interview and often in a different session, and this correlation may be less subject to bias. These biases must be borne in mind in the interpretation of the results, but are an intrinsic part of self-reports of lived experience and it is precisely this subjectivity that is valuable in this context, particularly when it is able to complement more objective blind-rated measures.

The AFEQ was designed to measure parental priorities for key outcomes of an early intervention for their autistic child and family. An interesting question is whether the final questionnaire measures what we, as its developers, hoped it would. As researchers, we are confident that the items do indeed reflect parental priorities: all the questionnaire items were generated directly from discussions by parents in response to open questions designed to tap these priorities. However, there are two issues which we feel were under-represented within the questionnaire: the issue of sibling relationships and that of parental couple relationships. A number of themes related to siblings were raised within the focus groups. One item specifically about siblings had to be removed in the data preparation phase as it generated large amounts of missing data in families with only one child. Other items (e.g., “I feel guilty about not giving other members of the family enough attention”) were phrased in such a way that they *could* be interpreted as being about siblings in families who had more than one child, but did not ask *specifically* about sibling relationships. Future research might seek to develop a “sibling relationship” questionnaire which could be used alongside the AFEQ by families with more than one child. Another issue that may be under-represented within the questionnaire is that of parental couple relationships and how these are affected, both positively and negatively, by having a child with autism within the family. Parents in the focus groups did not provide any discussion around this issue, perhaps due to inhibitions around discussing this personal topic in a group of relative strangers. This is something that could also be addressed in future research; possibly one-to-one interviews with parents or written questionnaires would be more likely to elicit reflection on this topic.

In summary, the AFEQ holds promise as a viable, parent-nominated measure of family life, prioritised intervention outcomes and change indicators. It was developed in a sequential manner to be truly user-generated, shows ecological validity and is sensitive to change. It is not in itself intended as a formal Quality of Life Measure but might be used in future work alongside generic quality of life measures, to evaluate family experience and to test associations between family experience and other factors, such as demographic variables and parental mental health. It could be applied in a number of research, health care and developmental settings to quantify the experience of families of children with autism and similar neurodevelopmental disorders.

## Electronic supplementary material

Below is the link to the electronic supplementary material.

Supplementary material 1 (DOC 108 KB)
