# Supplementary material for: The Autism Family Experience Questionnaire (AFEQ): An Ecologically-Valid, Parent-Nominated Measure of Family Experience, Quality of Life and Prioritised Outcomes for Early Intervention
Source: J Autism Dev Disord. 2017 Nov 18;48(4):1052–62. doi: 10.1007/s10803-017-3350-7 (PMC5861155; doi:10.1007/s10803-017-3350-7)
Supplement: Supplementary file 1 — Supplementary material 1 (DOC 108 KB) [file 10803_2017_3350_MOESM1_ESM.doc]

**Appendix I**

**Autism Family Experience Questionnaire (AFEQ)**

**EXPERIENCE OF BEING A PARENT OF A CHILD WITH AUTISM**

|  |  | **Always** | **Often** | **Sometimes** | **Rarely** | **Never** | **N/A** |
| --- | --- | --- | --- | --- | --- | --- | --- |
| **No** | **Item** | **1** | **2** | **3** | **4** | **5** |  |
| 1 | I lack confidence in knowing how to help my child |  |  |  |  |  |  |
| 2 | I feel listened to by professionals |  |  |  |  |  |  |
| 3 | Working with therapists or professionals helps me feel confident |  |  |  |  |  |  |
| 4 | I am confident that I understand my child’s level of development |  |  |  |  |  |  |
| 5 | I feel I know how to help my child progress |  |  |  |  |  |  |
| 6 | I feel I’m getting it wrong |  |  |  |  |  |  |
| 7 | I have realistic milestones for my child’s development |  |  |  |  |  |  |
| 8 | I doubt my ability to help my child’s development |  |  |  |  |  |  |
| 9 | I feel frustrated at not knowing how to help my child |  |  |  |  |  |  |
| 10 | I have coping mechanisms to help my child |  |  |  |  |  |  |
| 11 | Professionals don’t understand my family’s needs |  |  |  |  |  |  |
| 12 | It’s a continual battle to get the right help for my child |  |  |  |  |  |  |
| 13 | My child is getting the right help |  |  |  |  |  |  |

**FAMILY LIFE**

|  |  | **Always** | **Often** | **Sometimes** | **Rarely** | **Never** | **N/A** |
| --- | --- | --- | --- | --- | --- | --- | --- |
| **No** | **Item** | **1** | **2** | **3** | **4** | **5** |  |
| 14 | Family life is a battle |  |  |  |  |  |  |
| 15 | I feel guilty about not giving other members of the family enough attention |  |  |  |  |  |  |
| 16 | My child is flexible in adapting to the demands of family life |  |  |  |  |  |  |
| 17 | Family life is calm |  |  |  |  |  |  |
| 18 | I know how to cope with my child when going on an outing in a public place e.g. café or restaurant |  |  |  |  |  |  |
| 19 | I feel confident to go out to family events with my child |  |  |  |  |  |  |
| 20 | I feel confident in making routines at home more manageable for my child |  |  |  |  |  |  |
| 21 | I feel comfortable about having visitors to our home |  |  |  |  |  |  |
| 22 | My child has fussy eating that makes it difficult to go away for a break |  |  |  |  |  |  |

**CHILD DEVELOPMENT, UNDERSTANDING AND SOCIAL RELATIONSHIPS**

|  |  | **Always** | **Often** | **Sometimes** | **Rarely** | **Never** | **N/A** |
| --- | --- | --- | --- | --- | --- | --- | --- |
| **No** | **Item** | **1** | **2** | **3** | **4** | **5** |  |
| 23 | My child can concentrate on an activity for a short time |  |  |  |  |  |  |
| 24 | My child can spontaneously begin communication with me |  |  |  |  |  |  |
| 25 | My child spontaneously begins communication with other members of the family |  |  |  |  |  |  |
| 26 | My child can request his/ her needs appropriately |  |  |  |  |  |  |
| 27 | My child gets frustrated at not being understood |  |  |  |  |  |  |
| 28 | My child can let me know when he/ she is hurt |  |  |  |  |  |  |
| 29 | I know when my child feels poorly |  |  |  |  |  |  |
| 30 | My child has repetitive behaviour and sensory interests that make it difficult to go on an outing |  |  |  |  |  |  |
| 31 | My child is good at sharing with others |  |  |  |  |  |  |
| 32 | My child has to have his/ her own way |  |  |  |  |  |  |
| 33 | My child is aware of other people’s needs |  |  |  |  |  |  |
| 34 | My child gets invited to birthday parties |  |  |  |  |  |  |
| 35 | My child plays with other children |  |  |  |  |  |  |
| 36 | I have to go with my child to supervise play with other children |  |  |  |  |  |  |

**CHILD SYMPTOMS (FEELINGS AND BEHAVIOUR)**

|  |  | **Always** | **Often** | **Sometimes** | **Rarely** | **Never** | **N/A** |
| --- | --- | --- | --- | --- | --- | --- | --- |
| **No** | **Item** | **1** | **2** | **3** | **4** | **5** |  |
| 37 | My child is happy |  |  |  |  |  |  |
| 38 | My child is anxious |  |  |  |  |  |  |
| 39 | My child is tolerant of mistakes |  |  |  |  |  |  |
| 40 | My child is calm |  |  |  |  |  |  |
| 41 | My child is angry |  |  |  |  |  |  |
| 42 | My child is unpredictable |  |  |  |  |  |  |
| 43 | My child can let me know what he/she is upset about |  |  |  |  |  |  |
| 44 | My child understands appropriate behaviour in familiar social situations |  |  |  |  |  |  |
| 45 | My child knows the difference between family members and strangers |  |  |  |  |  |  |
| 46 | My child acts differently with family members compared with strangers |  |  |  |  |  |  |
| 47 | My child is embarrassing when going out |  |  |  |  |  |  |
| 48 | My child has repetitive behaviours that make day to day life impossible |  |  |  |  |  |  |
